# In-Fiber BaTiO_3_ Microsphere Resonator for High-Sensitivity Temperature Measurement

**DOI:** 10.3390/mi12030318

**Published:** 2021-03-18

**Authors:** Chi Li, Meng Zhu, Peng Ji, Cong Xiong, Changrui Liao

**Affiliations:** 1 Key Laboratory of Optoelectronic Devices and Systems of Ministry of Education, College of Physics and Optoelectronic Engineering, Shenzhen University, Shenzhen 518060, China; 2170285316@email.szu.edu.cn (C.L.); 1800282047@email.szu.edu.cn (M.Z.); jipeng@szu.edu.cn (P.J.); xiongcong2018@email.szu.edu.cn (C.X.); 2 Shenzhen Key Laboratory of Photonic Devices and Sensing Systems for Internet of Things, Guangdong and Hong Kong Joint Research Centre for Optical Fibre Sensors, Shenzhen University, Shenzhen 518060, China

**Keywords:** whispering gallery mode, fiber, resonators, temperature sensor

## Abstract

A fiber optic whispering gallery mode (WGM) resonator was proposed and realized by integrating an inline polymer waveguide with a microsphere mounted on it. The polymer waveguide with a diameter of 1 μm was printed with femtosecond laser-assisted multiphoton polymerization in a section of a grooved hollow-core fiber, which was sandwiched between two single-mode fibers. Two WGW resonators assembled with microspheres of different sizes were prepared. The transmission spectra of those stimulated WGMs were investigated both in simulation and experimentally. The temperature response of the resonators was particularly studied, and a linear sensitivity of −593 pm/°C was achieved from 20 °C to 100 °C.

## 1. Introduction

Whispering gallery mode (WGM) resonators have been widely applied in various fields, such as silicon photonic devices, biosensing, and optical frequency combs [1,2,3,4]. For a typical WGM resonator, a micro-/nano-ring is usually manufactured by a focused ion beam etching technique, which has the advantage of obtaining an ultra-high Q factor [5,6]. For example, a high Q factor (Q > 20,000) microresonator was fabricated to image nanophotonic modes by employing focused ion beam etching technology [7]. In addition, a ring resonator (Q > 30,000) for realizing a high-performance microdisk laser was successfully prepared with the same method [8]. However, the development of additive manufacturing technology, especially the femtosecond laser (fs-laser)-assisted multiphoton polymerization technique, has aroused an increasing interest to explore new ways to prepare WGM resonators in a more flexible manner [9,10].

In 2017, Hou et al. fabricated a polymer microsphere by multiphoton polymerization to create an optical laser with single-mode output [11]. Subsequently, in 2019, Kelemen et al. fabricated a polymer ring resonator connecting two optical fibers to detect reflective index changes of ambient biomass [12]. In this paper, we propose an all fiber-integrated WGM resonator, exhibiting an ultracompact configuration of 150 μm long and a high Q factor of Q_max_ = 10^3^. For the proposed WGM resonator, fs-laser-assisted multiphoton polymerization was employed to fabricate an inline polymer waveguide to guide the light beam. A barium titanate microsphere was mounted on the polymer waveguide to stimulate WGMs when the coupling mode conditions were met. Two WGW resonators assembled with microspheres of different diameters of 20 μm and 50 μm were fabricated to study the spectral characteristics and temperature response. For comparison, a finite difference time domain (FDTD) method was used to calculate the mode profile and the corresponding transmission spectrum. The ultracompact configuration has potential applications in detection with limited space or in an optical fiber-integrated system.

Due to the significant thermo-optical effect of barium titanate material [13,14], the temperature sensitivity of the proposed WGM resonator has been tested to −593 pm/°C, which is about 60 times that of traditional fiber optic temperature sensors, such as fiber Bragg grating-based devices [15,16]. Furthermore, various microsphere materials, such as antibody assemble sphere, SiC sphere, and optical Kerr effect sphere, present a potential capacity to realize multifunctional WGM resonators [2,3,17].

## 2. Materials and Methods

**Figure 1:**
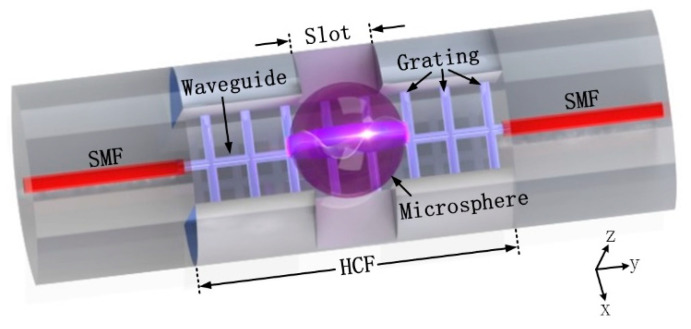
Schematic diagram of the polymer fiber-integrated whispering gallery mode (WGM) resonator.

Figure 1 shows the schematic diagram of the proposed polymer fiber-integrated WGM resonator. A polymer waveguide with a series of grating structures was embedded in a section of a grooved silica hollow-core fiber (HCF), which was spliced between two single-mode fibers (SMFs). The polymerized grating structure was designed to enhance the overall structural stability and keep the polymer waveguide in suspension to increase the optical evanescent field in the air. Additionally, a pair of physical slots in the x-direction was constructed to lock the microsphere within the fiber. WGMs were stimulated after mounting a smooth microsphere on the polymer waveguide. Therefore, the light beam with a specific wavelength transmitted through the suspended polymer waveguide was partially coupled into the microsphere when the phase-matching condition was reached.

**Figure 2:**
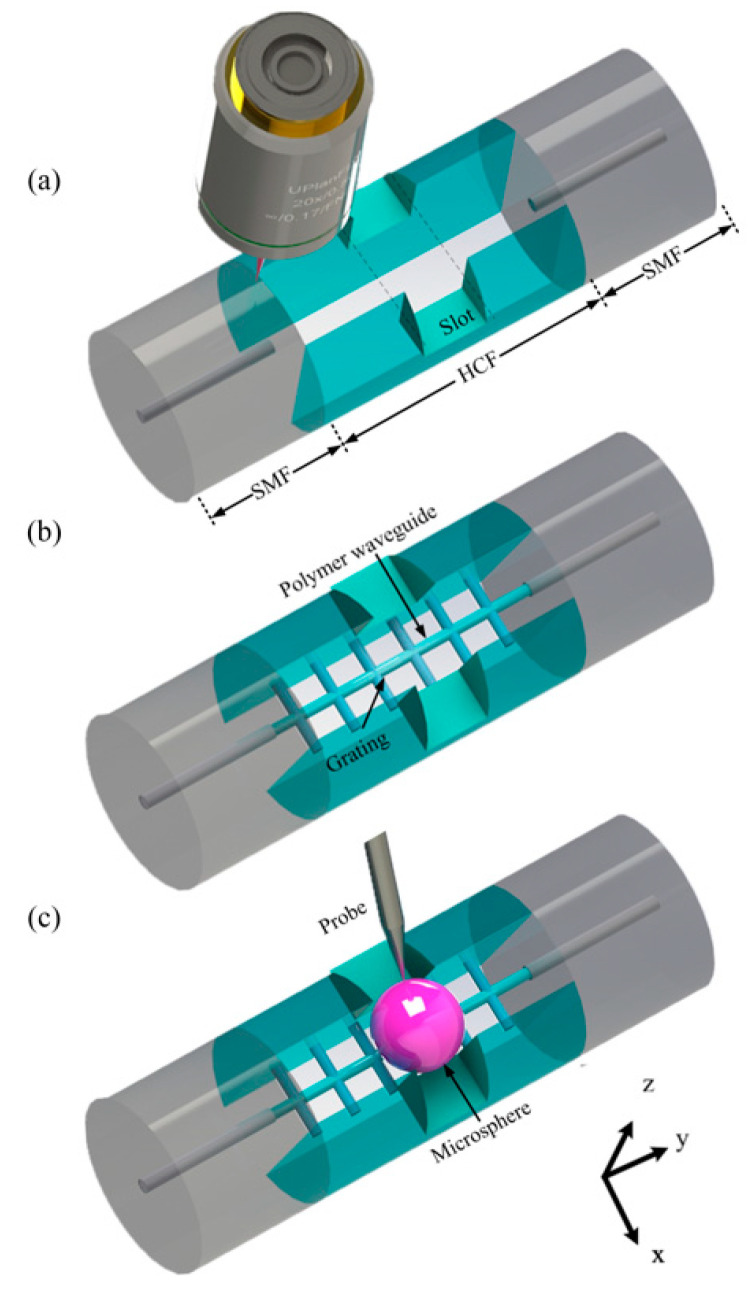
Fabrication procedure for the proposed WGM resonator. (**a**) A fs-laser was employed to realize subtractive manufacturing (ablation) after a section of hollow-core fiber (HCF) spliced between two single-mode fibers (SMFs). (**b**) A polymer waveguide was printed by additive manufacturing (polymerization) assisted with a fs-laser to connect the two SMFs. The grating segment was similarly printed. (**c**) A microsphere was mounted upon the polymer waveguide to generate WGM resonance.

The fabrication procedure is illustrated in Figure 2 and is similar to our previous work [18]. First, a section of HCF with a length of 150 μm was accurately cut out and spliced between two SMFs by an optical fiber fusion splicer (FUJIKURA 80 S) with an optimized parameter (splicing current of −10 bit for 400 ms). Commercially available HCF and SMF were adapted in this study, for which the internal/external and core/cladding were 15/125 μm and 5.8/125 μm in diameter, respectively. A fs-laser with a center wavelength of 800 nm and a repetition rate of 1 kHz was then employed to drill a throughout groove in the z-direction and a pair of slots in the x-direction within the HCF, as presented in Figure 2a. After laser ablation, an ultrasonic cleaner was used to wash out the debris remaining in the HCF.

After the grooved HCF was fully filled with liquid photoresist, the sample was mounted on a 3D air-bearing stage for fs-laser-assisted polymerization. For the fs-laser system, the center wavelength, repetition rate, and pulse duration were 1026 nm, 220 kHz, and 250 fs, respectively. The polymer waveguide and grating structures were printed using a 63× oil objective lens with NA = 1.4. During the printing process, the scanning speed and laser intensity were controlled to 200 μm/s and 5.82 × 10^12^ W/cm^2^, respectively. After being immersed in a mixture of acetone and isopropanol (volume ratio: 1:3) for 20 min, the uncured liquid photoresist was washed away, while the desired structure was preserved in the HCF, as shown in Figure 2b.

After the ethanol was completely volatilized, a commercially available barium titanate microsphere was carefully mounted on the polymer waveguide through a tungsten probe, as shown in Figure 2c. 

## 3. Results and Discussion

Two microspheres with different diameters were prepared to study the WGM resonance. The transmission spectrum of the proposed WGM resonator was experimentally explored with an optical spectrum analyzer (OSA, YOKOGAWA, AQ6370C, Tokyo, Japan) and a broadband light source ranging from 1250 nm to 1650 nm.

**Figure 3:**
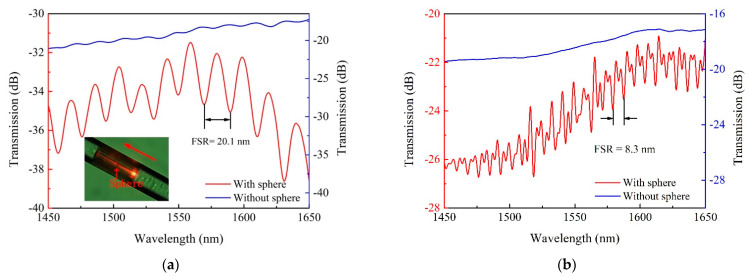
Detected transmission spectra before and after mounting a microsphere with a diameter of (**a**) 20 μm and (**b**) 50 μm.

When a visible light with a wavelength of 650 nm was tentatively illuminated on the WGM resonator, as indicated in the inset of Figure 3a, the input light was evidently observed to be guided through the polymer waveguide, and the coupled light caused by the resonance effect appeared near the microsphere. Additionally, significant scattering loss also occurred at the interface between the polymer waveguide and the SMF.

As seen in Figure 3 in the detected output signal, the red line and the blue line represent the transmission spectra of the polymer waveguide with and without a microsphere mounted on, respectively. An insertion loss of about 10 dB occurs, which may be caused by the deformation of the polymer waveguide. For samples equipped with microspheres with diameters of 20 ± 2 μm and 50 ± 2 μm, the free spectral range (FSR) was measured as 20.1 nm and 8.3 nm, respectively. The FSR before and after the measured values were 20.0 nm, 20.1 nm, and 20.7 nm, and 7.8 nm, 8.3 nm, and 7.0 nm, and their standard errors were 0.4 nm and 0.6 nm, respectively, so the FSR measured was 20.1 ± 0.4 nm and 8.3 ± 0.6 nm, respectively. Various factors (e.g., machining error, laser energy fluctuation) may affect the insertion loss even for the same fabrication procedure.

**Figure 4:**
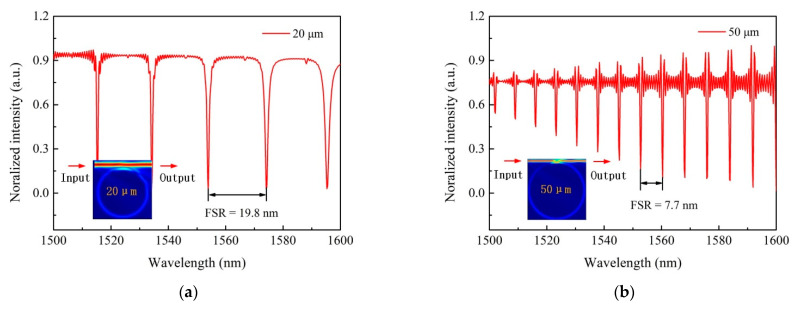
Calculated transmission spectra of different WGM resonators for microspheres with a diameter of (**a**) 20 μm and (**b**) 50 μm.

A finite difference time–domain software (Mode solution) was employed to calculate the transmission spectrum of the fiber-integrated WGM resonator. The finite difference time–domain method (FDTD) directly discretizes the time–domain wave equation without any form of derived equation, so its application scope will not be limited by the mathematical model. Its difference scheme contains the parameters of the medium, and it can simulate all kinds of complex structures by being given only the corresponding parameters to each grid. This is an outstanding advantage of the FDTD method [19]. Figure 4a,b shows the calculated results of two WGM resonators with microsphere diameters of 20 μm and 50 μm (reflective index of 1.86 at 1550 nm), as well as the corresponding mode profiles. The calculated FSRs are 19.8 nm and 7.7 nm, respectively, which are in good agreement with the above experimental results. The FSR can be expressed as [20]:

(1) $$\mathrm{FSR}=\frac{\lambda^{2}}{2\pi \mathrm{nr}}\;$$

where λ is the coupled wavelength, and n and r are the refractive index and radius of the microsphere, respectively.

**Figure 5:**
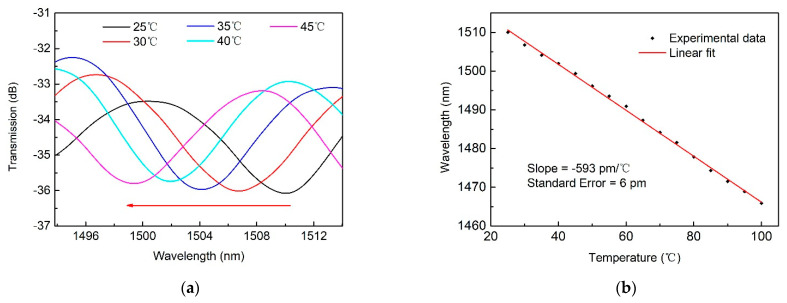
(**a**) Transmission spectra for the WGM resonator under different ambient temperatures for the case of a 20 μm microsphere. (**b**) Linear fit of the measured temperature response.

The temperature response of the proposed WGM resonator was tested with a sample equipped with a microsphere with a diameter of 20 μm. During the test, the sample was enclosed in a controllable furnace, and a specific resonance wavelength of 1510.5 nm was used to monitor changes in ambient temperature. The temperature was gradually increased from 25 °C to 100 °C in steps of 5 °C, maintaining each step for 20 min. As depicted in Figure 5a, a blue shift of the resonance wavelength was observed. The linear fit of its temperature response was plotted in Figure 5b, giving rise to a sensitivity of −593 pm/°C with a standard error of 6 pm. Note that the sensitivity achieved is approximately 60 times that of traditional fiber optic temperature sensors, such as fiber Bragg gratings.

According to the well-known principle of temperature sensing, the relationship between the wavelength shift (∆λ_0_) at the wavelength λ_0_ and the temperature change ∆T can be given by [21]:

(2) $$\frac{\Delta \lambda_{0}}{\Delta T}=\left(\alpha +\xi \right)\lambda_{0},$$

where α and ξ are the thermo-expansion coefficient and the thermo-optic coefficient, respectively. In general, the thermal photonic effect dominates the wavelength shift because crystal materials scarcely have a volumetric change as temperature deviation. As for the BaTiO_3_ particle, the electric permittivity has a dramatic change in the size difference from nanometer to micrometer [14,22,23,24,25]. The thermal expansion coefficient of barium titanate is about 10^‒6^/°C [26,27], which is two orders of magnitude smaller than the thermo-optic coefficient, so the size change caused by temperature is negligible.

## 4. Conclusions

In sum, a polymer fiber-integrated WGM resonator was embodied with the help of fs-laser-assisted polymerization and micromanipulation technology, possessing an ultracompact configuration with a length of 150 μm and a high Q factor of Q_max_ = 10^3^. Q = λ/Δλ, where λ and Δλ denote the resonant wavelength and the full width at half-maximum (FWHM) of the wavelength, respectively [12]. The measured results including FSR, mode profile, and temperature sensitivity are in good agreement with the experimental results. The temperature sensitivity achieved by the proposed WGW resonator is −593 pm/°C, which is much higher than that of the traditional optical fiber temperature sensor. Reflective index deviation and the volumetric expansion of the polymer waveguide and the microsphere caused by temperature evolution are both able to modulate the coupling condition, which would change the resonant wavelength. Thus, how to precisely distinguish the temperature effect on the polymer waveguide and the microsphere might be best examined with a multiphysics software simulation model. By using different microspheres, such devices can be used in various fields, especially in biosensing and cell detection. In addition, the configuration of fiber-integrated WGM resonators is a potential and meaningful research topic [28,29,30].

## Data Availability

Not applicable.
